# Xanthomicrol Exerts Antiangiogenic and Antitumor Effects in a Mouse Melanoma (B16F10) Allograft Model

**DOI:** 10.1155/2020/8543872

**Published:** 2020-12-22

**Authors:** Foad Ghazizadeh, Massoumeh Shafiei, Reza Falak, Mahshid Panahi, Naser Rakhshani, Soltan Ahmed Ebrahimi, Parvaneh Rahimi-Moghaddam

**Affiliations:** ^1^Department of Pharmacology, School of Medicine, Iran University of Medical Sciences, Hemmat Highway, Tehran 14496, Iran; ^2^Department of Immunology, School of Medicine, Iran University of Medical Sciences, Hemmat Highway, Tehran 14496, Iran; ^3^Department of Pathology, Firoozgar Hospital, Iran University of Medical Sciences, Tehran 15937, Iran

## Abstract

Xanthomicrol, a trimethoxylated hydroxyflavone, is the main active component of *Dracocephalum kotschyi* Boiss leaf extract. Preliminary in vitro studies identified this compound as a potential antiangiogenic and anticancer agent. This study aimed to evaluate in vivo anticancer effect of xanthomicrol and investigate its molecular mechanism of action in a mouse melanoma (B16F10) model. Effect of xanthomicrol on B16F10 melanoma cell viability was determined using the MTT assay. For in vivo experiments, C57BL/6 mice were inoculated subcutaneously with B16F10 cells. After five days, once daily administration of xanthomicrol, thalidomide, or vehicle was commenced and continued for 21 consecutive days. On the 26th day, blood samples and tumor biopsies were taken for subsequent molecular analysis. Xanthomicrol showed inhibitory effect on viability of B16F10 melanoma cells (IC50 value: 3.433 *μ*g/ml). Initial tumor growth, tumor volume and weight, and angiogenesis were significantly decreased in xanthomicrol-treated animals compared with those in vehicle group. Protein expression of phosphorylated Akt, mRNA expressions of HIF-1*α* and VEGF in tumor tissues, and serum VEGF were significantly decreased in xanthomicrol-treated animals compared with vehicle-treated animals. Thus, xanthomicrol inhibited cancer cell growth both in vitro and in vivo. This effect, at least in part, was exerted by interfering with PI3K/Akt signaling pathway and inhibiting VEGF secretion by tumor cells. Further studies are required to elucidate the exact molecular mechanisms of antitumor activity of xanthomicrol.

## 1. Introduction

Melanoma is an aggressive cancer of melanocytes with high morbidity and mortality. Apart from surgery in patients with early-diagnosed disease, very few satisfactory treatment options are available [1, 2]. Melanoma is a hypervascular tumor likely to produce metastases [3]. Traditional chemotherapy, together with radiation therapy, has not been successful in producing remission in metastatic melanomas. This has led researchers to look for alternative therapeutic options, which include targeted therapies and immunomodulators. Our increasing understanding of the role of the immune system in tumor development has prompted research into agents that can modulate the activity of the immune cells. However, evidence has been accumulated that shows a role for the immune cells in “cancer-immunoediting,” which can lead to the development of escape routes for melanoma tumor cells [2]. In order to mitigate the development of immunotolerance to malignant cells during the treatment of melanoma, use of combination therapy, that is, targeting multiple pathways, has been proposed. As angiogenesis is of great importance in the process of metastases generation, inhibiting de novo vascularization can be used as an adjunct therapy in the treatment of melanomas.

Inhibiting angiogenesis is a promising cancer therapeutic strategy that was first proposed by Judah Folkman [4]. Melanoma is a highly vascular tumor, and angiogenesis is a clear feature of cutaneous melanoma that is confirmed in preclinical models [5, 6]. Vascular endothelial growth factor (VEGF) is the most relevant mediator of tumor-associated angiogenesis, and its evaluation at both mRNA and protein levels has confirmed its correlation with disease progression and poor clinical outcome [7–9]. Overproduction of VEGF along with its receptor expression induces proliferation and survival of melanoma cells via mitogen-activating protein kinase (MAPK)/extracellular signal-regulated kinases (Erk) and phosphatidylinositol 3′-kinase (PI3K)/protein kinase B (Akt) signaling pathways [10]. It is worth mentioning that simultaneous activation of these two pathways is required for melanoma progression [11, 12]. It is also known that a major part of the action of these two pathways, in tumor angiogenesis and development, is mediated through increasing hypoxia-inducible factor 1-alpha (HIF-1*α*) transcription, which is the key mediator involved in VEGF secretion in hypoxic microenvironment [13–15]. The aforementioned findings make these molecules valuable targets for antiangiogenic research.

**Figure 1:**
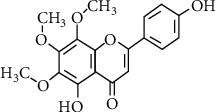
Chemical structure of xanthomicrol.

Phytochemicals have been extensively studied not only for improving the outcomes of the application of common chemotherapeutic regimens but also for potential use in cancer prevention strategies. They are unique compounds which can broadly interfere with diverse crucial features of pathological processes including tumor vascularization [16, 17]. Xanthomicrol (4′, 5-dihydroxy-6, 7, 8-trimethoxyflavone; Figure 1) is the main active component of *Dracocephalum kotschyi* Boiss leaf extract [18], a part of an Iranian traditional herbal remedy known as Spinal-Z, which has been used for treatment of some types of cancer [19]. Preliminary in vitro studies in our laboratory, using a number of cancer cell lines and human fetal foreskin fibroblast (HFFF-P16), as normal cells, showed that this compound could strongly inhibit preferentially and selectively the growth of malignant cells [20]. Further investigation revealed that xanthomicrol possessed profound antiangiogenic activity [21]. Preliminary pharmacokinetic and toxicological studies in mice demonstrated its usability and safety in vivo [22] and had allowed some workers to propose xanthomicrol as an anticancer candidate.

This study was undertaken to explore the effects of xanthomicrol on a melanocyte cancer cell line and then go on to investigate antitumor activity of xanthomicrol in a melanoma-bearing mouse model. This fundamental information will also shed light on the, as yet, poor understanding of the mechanism of antitumor activity of this plant-derived compound.

## 2. Materials and Methods

### 2.1. Drugs and Chemicals

Xanthomicrol was purchased from Angene Chemical Co. (Nanjing, China), and thalidomide was obtained from Symed Labs (Telangana, India). 3-[4, 5-Dimethylthiazol-2-yl]-2, 5 diphenyl tetrazolium bromide (MTT) and sodium carboxymethyl cellulose (CMC) were obtained from Sigma-Aldrich Chemical Co. (St. Louis, MO, USA). RPMI-1640, antibiotic-antimycotic, and trypsin-EDTA were supplied from Bioidea Co. (Tehran, Iran). Fetal bovine serum (FBS) was purchased from Gibco (life technologies, USA). Dimethyl sulfoxide (DMSO) was purchased from Merck (Germany).

### 2.2. Cell Culture and Cell Viability Assay

The HFFF-P16 (human fetal foreskin fibroblast) and B16F10 mouse melanoma cell lines were obtained from Pasteur Institute (Tehran, Iran). The cells were maintained in RPMI-1640 supplemented with 10% (v/v) heat-inactivated FBS, 100 units/ml penicillin, and 100 *μ*g/ml streptomycin at 37°C in a humidified atmosphere of 5% CO_2_. MTT assay was used to determine the viability of HFFF-P16 cells treated with xanthomicrol [20] and B16F10 cells treated with xanthomicrol or thalidomide [23]. HFFF-P16 cells (10,000 cells/well) and B16F10 cells (5000 cells/well) were seeded on 96-well culture plates and incubated under 5% CO_2_ air at 37°C for 24 h (confluency >80%, passage number 2–3).

For HFFF-P16 cells, the culture medium was then replaced with 200 *μ*l fresh medium containing vehicle (0.2% DMSO) or xanthomicrol (5, 10, 25, 50, and 100 *μ*g/ml). For B16F10 cells, the culture medium was replaced with 200 *μ*l serum-free medium containing vehicle (0.2% DMSO), xanthomicrol (0.5, 1, 2.5, 5, 10, 25, 50, and 100 *μ*g/ml), or thalidomide (1, 5, 10, 50, 100, 250, and 500 *μ*g/ml). After incubation for 72 h (for HFFF-P16 cells) or 48 h (for B16F10 cells) at 37°C and 5% CO_2_, 20 *μ*l of MTT solution (5 mg/ml in deionized water) was added to each well and incubated for 3 h under the same condition. The medium was aspirated thereafter, and the resulting intracellular purple formazan was solubilized in 100 *μ*l DMSO and quantified at 570 nm using a Dynex MRX microplate reader. Cell viability was determined by dividing the absorbance readings from test samples by those of the control. IC_50_ value was defined as the drug concentration that is required for 50% inhibition of cell proliferation and calculated using GraphPad Prism software, version 6.07, USA.

### 2.3. Animals

Female C57BL/6 mice (6–8 weeks old; 20–25 g body weight) were obtained from Pasteur Institute (Tehran, Iran). All animals were kept under controlled conditions with 12 h light/12 h dark cycle and had free access to food and water. Animal care and all experiments were performed in accordance with the National Institutes of Health Guide for the Care and Use of Laboratory Animals (NIH Publications no. 8023, revised 1978) and approved by the Ethics Committee of Iran University of Medical Sciences (Ethical code: IR.IUMS.REC.FMD.1395.24121).

### 2.4. In Vivo Antitumor Experiments

B16F10 melanoma cells (10^5^ cells in 100 *μ*l PBS) were injected subcutaneously into the right flanks of C57BL/6 mice (*n* = 30). Five days after tumor inoculation, thirty mice were divided randomly into three groups (10 mice in each group): vehicle control group (CMC 0.5%), positive control group (thalidomide 200 mg/kg), and test group (xanthomicrol 50 mg/kg). Thalidomide and xanthomicrol were dissolved in 0.5% CMC at final concentrations of 25 mg/ml and 6.25 mg/ml, respectively. Drug administration was performed intraperitoneally (0.2 ml) once a day for a total of 21 consecutive days [24, 25]. Tumor diameters were measured every other day after becoming palpable. Tumor volumes were calculated using the following formula: (length × width^2^)/2 [25]. Animals were weighed twice a week and observed for motor/behavioral abnormalities. On the 26^th^ day after tumor inoculation, animals were first anaesthetized with ketamine (100 mg/kg)/xylazin (10 mg/kg), and blood samples were collected by cardiac puncture. Then, mice were immediately sacrificed by CO_2_ asphyxiation and tumor tissues were harvested and weighed. A portion of each tumor sample was stored at -80°C for quantitative real-time PCR and western blot analysis, while the rest was fixed in 10% neutral buffered formalin and embedded in paraffin for subsequent immunohistochemical assessments. Also, liver, spleen, kidney, and lungs were removed, fixed in 10% neutral buffered formalin, embedded in paraffin, and examined histologically, in a blind fashion, after hematoxylin staining. Serum was also separated from whole blood and used for ELISA analysis.

### 2.5. Immunohistochemical Staining and Counting

Immunohistochemical staining was carried out according to a previously published procedure [26]. Briefly, formalin-fixed, paraffin-embedded tumor tissue sections (3–4 *μ*m) were prepared on charged slides. Staining procedure was performed using Master Polymer Plus Detection System (Master Diagnostica, Granada) using an anti-CD31 mouse monoclonal antibody (sc-376764; Santa Cruz Biotechnology, Santa Cruz, CA, USA; dilution 1 : 50). Immunostaining visualization was performed using 3, 3′-diaminobenzidine (DAB) as the chromogen. Sections were counterstained with hematoxylin. Microvessel density (MVD) was determined semiquantitatively by counting the CD3-positive vessels in at least five areas of highest vascularity, called “hotspots” in randomly selected sections.

### 2.6. Quantitative Real-Time PCR

Total RNA was extracted from tumor tissues with ONE STEP-RNA Reagent (Bio Basic Inc., Canada). Quantity and purity of isolated RNA was assessed spectrophotometrically using 260/280 nm absorbance ratio. Complementary DNA (cDNA) was prepared from 1 *μ*g RNA sample using RevertAid^TM^ First Strand cDNA Synthesis Kit (Thermo Fisher Scientific Inc., USA) according to the manufacturer's protocol. 1 *μ*l aliquots of cDNA samples were amplified by 2X SYBER Green PCR Master Mix (BioFACT^TM^, Korea), combined with 10 pmol of forward and 10 pmol of reverse primer for different primers of mouse gene (Table 1) in a final volume of 20 *μ*l. Real-time reactions were run in duplicate on a Rotor-Gene^®^ Q system (Qiagen) using the following cycling protocol: 15 minutes at 95°C followed by 40 cycles of 20 seconds each at 95°C, 20 seconds at 60°C, and 20 seconds at 72°C. Quantitative analyses of C_t_ values were done using Rotor-Gene Q Series software version 2.3.1 (Qiagen), and relative expression levels were normalized to HPRT1 expression (as housekeeping gene) using the 2 (ΔΔC_t_) formula.

### 2.7. ELISA

A highly specific quantitative sandwich ELISA kit for mouse VEGF was purchased from R&D Systems (Minneapolis, Minn., USA), and serum VEGF level was evaluated according to manufacturer's instructions.

### 2.8. Western Blot Analysis

Protein extraction was performed by homogenizing tumor tissues in lysis buffer (sc-24948; Santa Cruz Biotechnology). Protein concentrations were quantified using bicinchoninic acid (BCA) assay kit (Parstous Company, Tehran, Iran). Western blot analysis was done as previously described [21]. Extracted proteins were separated using 15% sodium dodecyl sulfate polyacrylamide gel (SDS–PAGE). Protein bands were blotted onto polyvinylidene difluoride (PVDF) membranes. Immunostaining was carried out by blocking PVDF membranes in tris-buffered saline containing 0.1% v/v Tween 20 (TBST) and 5% non-fat dry milk, followed by probing using anti-Akt (sc-81434), anti-phospho-Akt (sc-81433), anti-Erk (sc-135900), anti-phospho-Erk (sc-81492), or anti-*β*-actin (sc-47778) antibodies (all purchased from Santa Cruz Biotechnology, USA; diluted in TBST and 2% non-fat dry milk; dilution 1 : 100). Immunoblots were then incubated with anti-mouse IgG antibody conjugated to horseradish peroxidase (sc-516102; Santa Cruz Biotechnology, USA; diluted in TBST and 2% non-fat dry milk; dilution 1 : 1000). The bands were detected using enhanced chemiluminescence detection kit (Parstous Company, Tehran, Iran) and analyzed with Fusion^®^ imaging system (Vilber Lourmat, France).

### 2.9. Statistical Analysis

All data were presented as mean ± standard error of mean (SEM). Statistical tests were performed using one-way ANOVA and Tukey's post-hoc test (GraphPad Prism software, version 6.07, USA). Two-way ANOVA was used to compare tumor volumes of different groups. *P* < 0.05 was considered to be significantly different.

## 3. Results

### 3.1. Xanthomicrol Attenuated Viability of B16F10 Melanoma Cells

**Figure 2:**
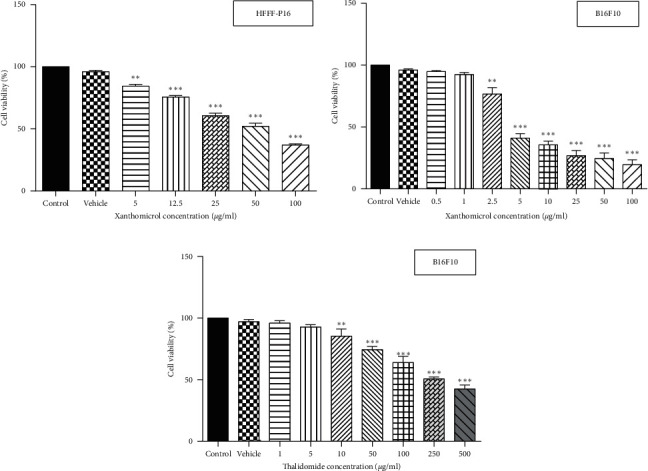
Xanthomicrol decreased viability of B16F10 melanoma cells. HFFF-P16 treated with vehicle (0.2% DMSO) and xanthomicrol (a). Serum-starved B16F10 cells were treated with vehicle (0.2% DMSO), xanthomicrol (b), or thalidomide (c) for 48 h, and cell viability was determined by the MTT assay. Data are represented as mean ± SEM (^∗∗^*P* < 0.01, ^∗∗∗^*P* < 0.001 vs. vehicle group).

MTT assay was used to assess the effect of xanthomicrol on viability of HFFF-P16 cells (IC_50_ value: 53.69 *μ*g/ml) (Figure 2(a)). Effects of xanthomicrol and thalidomide on viability of B16F10 cells were also determined, and as shown in Figures 2(b) and 2(c), both compounds exhibited a concentration-dependent antiproliferative activity after 48h exposure. Xanthomicrol showed statistically significant (*P* < 0.01) reduction in cell viability at concentrations equal or higher than 2.5 *μ*g/ml (IC_50_ value: 3.433 *μ*g/ml) (Figure 2(b)), while thalidomide induced significant (*P* < 0.01) decrease in cell viability at concentrations equal or higher than 10 *μ*g/ml (IC_50_ value: 178.3 *μ*g/ml) (Figure 2(c)).

### 3.2. Xanthomicrol Retarded Tumor Growth and Progression In Vivo

In order to evaluate the in vivo anticancer efficacy, the ability of xanthomicrol to inhibit cancer progression was examined in the mouse melanoma (B16F10) allograft model.

Animals in the vehicle-treated group developed tumor on days 15–21 after tumor cell inoculation, while animals in the thalidomide- and xanthomicrol-treated groups developed tumors on days 15–23 and 15–25, respectively.

**Figure 3:**
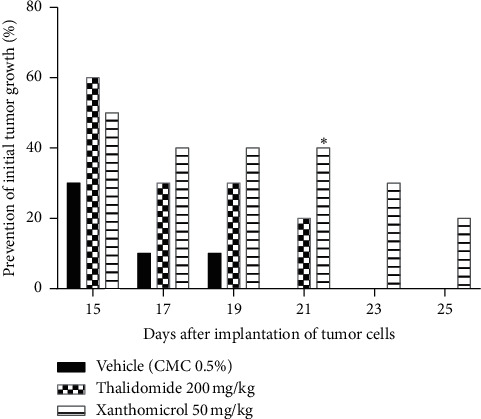
Xanthomicrol-retarded initial tumor growth in vivo. B16F10 melanoma cells were injected subcutaneously into the flanks of C57BL/6 mice. Treatments were initiated five days after tumor inoculation, once a day via i.p. route for 21 consecutive days. Animals in each group were observed for appearance of initial tumor and the percent of animals that did not show initial tumor in each day determined in experimental groups. Data are represented as mean ± SEM (^∗^*P* < 0.05 vs. vehicle-treated mice).

Both xanthomicrol and thalidomide showed inhibitory effects on initial tumor growth compared with vehicle. This inhibition became statistically significant only for xanthomicrol on the 21^th^ day after tumor cells implantation (Figure 3; *P* < 0.05). Importantly, in the cohort of animals treated with xanthomicrol, growth of subcutaneous B16F10 tumors was completely suppressed in two out of 10 mice until the end of the experiment (the 26^th^ day after inoculation).

**Figure 4:**
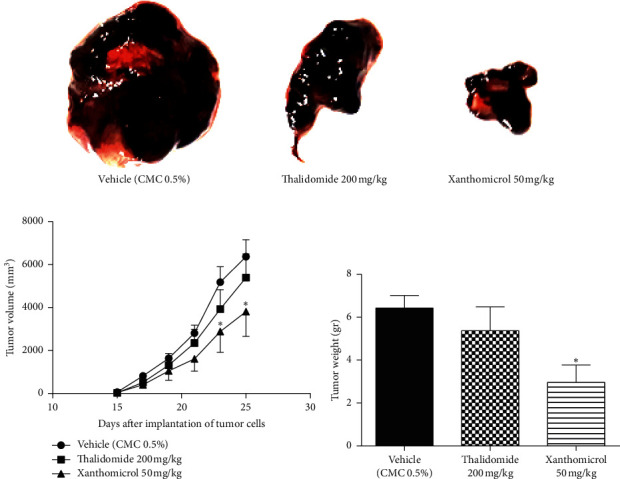
Xanthomicrol-retarded tumor progression in vivo. B16F10 melanoma cells were injected subcutaneously into the flanks of C57BL/6 mice. Treatments were initiated five days after tumor inoculation once a day via i.p. route for 21 consecutive days. Representative images of tumor mass in final day of the experiment (a). Tumor volumes were sized every other day (b), and tumor weights were measured after excisions of tumors in final day of the experiment (c). Data are represented as mean ± SEM (^∗^*P* < 0.05 vs. vehicle treated mice).

The melanoma tumors grew slower in xanthomicrol-treated animals than those in thalidomide and vehicle groups so that, by the 23^th^ day of tumor implantation, the tumor volume was significantly smaller in animals treated with xanthomicrol compared with tumors from untreated tumor-bearing mice (2880 ± 971.7 vs. 5187 ± 720.7 mm^3^; Figure 4(b); *P* < 0.05). At the end of the study, xanthomicrol-treated mice revealed on average a 59% reduction in tumor volume and a 46% reduction in tumor weight compared with control animals treated with vehicle (Figures 4(a)–4(c), resp.; *P* < 0.05).

### 3.3. Xanthomicrol Showed a Safe Anticancer Activity

In our experiments, we observed no signs of toxicity, including weight loss or behavioral abnormalities. We also found no histological damage to vital organs in drug-treated animals (data not shown).

### 3.4. Xanthomicrol Attenuated Tumor Angiogenesis In Vivo

As angiogenesis is a critical process for tumor progression, immunohistochemical CD31 staining was applied to assess tumor angiogenesis in excised B16F10 tumors after 21 consecutive days of treatment.

**Figure 5:**
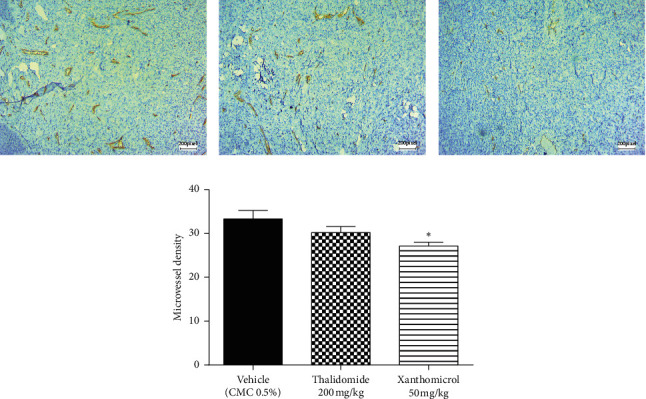
Xanthomicrol attenuated tumor angiogenesis in vivo. Representative images (((a) vehicle, (b) thalidomide, and (c) xanthomicrol) and quantification (d) of endothelial cells stained by CD31 (brown) on tumor sections of B16F10 tumors implanted subcutaneously in C57BL/6 mice after 21 days of treatment. Data are represented as mean ± SEM (^∗^*P* < 0.05 versus vehicle treated mice).

Figure 5 shows CD31 staining of tumor sections treated with vehicle (Figure 5(a)), thalidomide (Figure 5(b)), and xanthomicrol (Figure 5(c)). Xanthomicrol significantly (*P* < 0.05) reduced tumor MVD in tumor-bearing mice compared with vehicle control group (Figure 5(d)). Thalidomide also exhibited a decreasing trend of tumor MVD although it was not statistically significant in our experiment. This finding indicates that tumor angiogenesis was decreased by xanthomicrol treatment in subcutaneous mouse melanoma model.

### 3.5. Xanthomicrol Inhibited HIF-1*α*/VEGF Axis and Release of VEGF In Vivo

HIF-1*α* and its key transcriptional target, VEGF, are the main factors mediating tumor growth and angiogenesis. Circulating VEGF is also a valuable predictive marker for tumor progression and its response to anticancer therapies.

To assess the effects of xanthomicrol on these factors, tumor tissue and serum samples of animals in final day of treatment were analyzed using qRT-PCR and ELISA methods, respectively.

**Figure 6:**
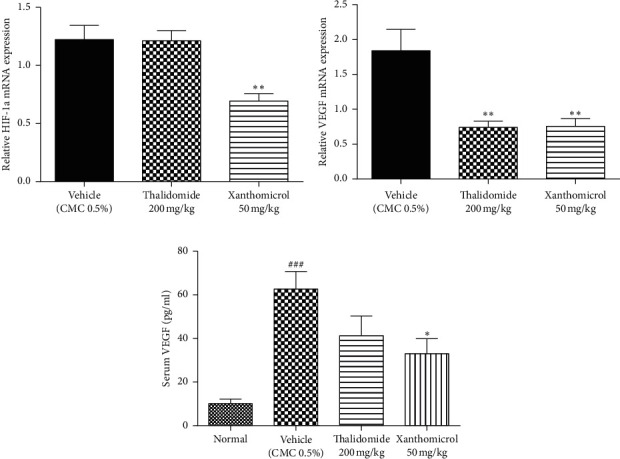
Xanthomicrol inhibited HIF-1*α*/VEGF axis and release of VEGF in vivo. After 21 consecutive days of treatment, animals were sacrificed, and tumor tissues were excised and used for quantitative RT-PCR analysis of HIF-1*α* (a) and VEGF (b) mRNA expression. HPRT1 expression was used as housekeeping gene. Blood samples were also collected by cardiac puncture and levels of serum VEGF were determined using an ELISA kit (c). Data are represented as mean ± SEM (^###^*P* < 0.001 versus normal, nontumor*-*bearing mice; ^∗∗^*P* < 0.01 and ^∗^*P* < 0.05 vs. vehicle-treated mice).

Figures 6(a) and 6(b) show that xanthomicrol significantly inhibited tumor-specific mRNA expression of both HIF-1*α* (*P* < 0.01) and VEGF (*P* < 0.01), respectively. These figures also indicate although thalidomide did not exert inhibitory effect on HIF-1*α* mRNA expression, it significantly reduced mRNA expression of VEGF (*P* < 0.01).

Figure 6(c) shows VEGF serum levels increased dramatically (*P* < 0.001) in vehicle-treated B16F10 allografted animals compared with normal, nontumor-bearing mice. Elevated levels of serum VEGF were significantly decreased in the group treated with xanthomicrol (*P* < 0.05). Thalidomide also showed a trend to reduce elevated levels of serum VEGF although it was not statistically significant in our experiment.

### 3.6. Xanthomicrol Decreased Akt Phosphorylation In Vivo

Erk and Akt are two key downstream effectors of MAPK and PI3K signaling pathways, respectively, both of which can control HIF-1*α*/VEGF axis in melanoma. To gain a better insight into the molecular mechanism of xanthomicrol action in melanoma, excised tumors of animals in final day of treatment were further assessed by western blotting for phosphorylation levels of these two key kinases.

**Figure 7:**
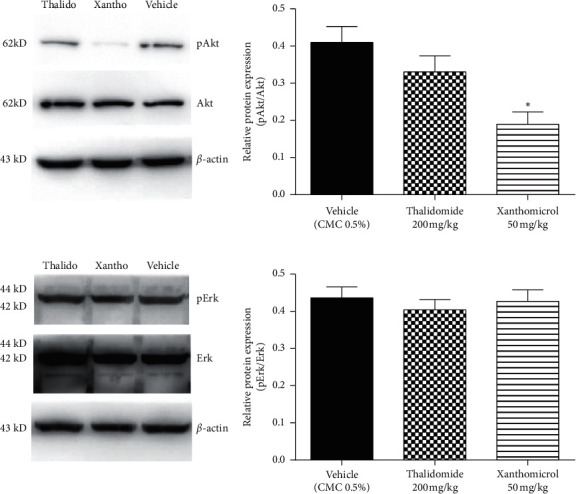
Xanthomicrol decreased Akt phosphorylation in vivo. After 21 consecutive days of treatment, excised tumor tissues from B16F10 allografted animals in different groups were used for protein extraction and subsequent western blot analysis of Akt, phosphorylated Akt (a), Erk, and phosphorylated Erk (b). *β*-Actin was used as loading control. Data are represented as mean ± SEM (^∗^*P* < 0.05 vs. vehicle-treated mice).

Xanthomicrol reduced the phosphorylation of Akt (Figure 7(a); *P* < 0.05), but not Erk (Figure 7(b)). Moreover, thalidomide caused a nonsignificant decrease in Akt phosphorylation (Figure 7(a)).

## 4. Discussion

Melanoma is a hypervascular neoplasm of melanocytes with high morbidity and mortality rates [3, 27]. Melanoma treatment using traditional chemotherapeutic agents has not been very successful, to the extent that melanoma is the cause of about 80% of all cutaneous carcinoma related deaths. Based on the presence of mutations in BRAF, RAS, or neurofibromatosis type 1 genes, the Cancer Genome Atlas Research (TCGA) network has recognized four geneticly distinct forms of melanoma. Agents have been developed that can interfere with the protein products of these genes and therefore halt the proliferation of malignant cells. These drugs include BRAF inhibitors such as dabrafenib and mitogen-activated protein kinase inhibitors like trametinib [28, 29]. Despite early optimism about the effectiveness of these agents in melanoma treatment, it appears that cancer cells become tolerant to the effects of these agents rather quickly. One strategy adopted to decrease the chances of tolerance development is using two or more of these selective proliferation inhibitors simultaneously [30]. In addition, workers have explored alternative/adjunct treatment options. These newer approaches include immunotherapy and use of antiangiogenic agents. A number of antibodies against various molecular targets have been developed and used alone or in combination for treatment of melanomas. These include ipilimumab (anti-CTLA-4 antibody) and nivolumab or pembrolizumab (anti-PD-1 antibodies). Although these agents, depending on the cancer type and other therapies used, can increase progression-free survival and overall survival, none has provided a major breakthrough.

One of the well-known agents recognized as having antiangiogenic activity is thalidomide [31]. Although this drug had been found to be a teratogen and unsuitable for its originally designed for application, it was later reintroduced as an anticancer agent mainly because of its antiangiogenic properties. Later on, it was found to modulate for function of the immune system as well [32]. Understanding the mechanisms involved in angiogenesis and the discovery that vascular endothelial growth factor (VEGF) appeared to have a central role has led to the development of anti-VEGF and anti-VEGF receptor (VEGFR) antibodies as anticancer agents, which have been used with varying degrees of success for the treatment of some cancers [33]. Our greater understanding of the pivotal role of receptor protein kinases (RPK) in the angiogenic process has led to the development of small molecule inhibitors of these ATP-dependent protein phosphorylating enzymes. Drugs such as sorafenib and sunitinib have found clinical application in the treatment of cancers, including melanoma [3]. Still, development of resistance to the antiangiogenic effects of these drugs has provided impetus for research into other antiangiogenic agents.

Xanthomicrol is an antiangiogenic agent, which has been proposed as a possible drug candidate for treatment of cancer [21]. Little is known about its in vivo anticancer efficacy or its exact molecular mechanism of action. This study was designed to investigate anticancer effects of xanthomicrol compared with thalidomide, one of the first small molecule antiangiogenic agents.

Results of our work and previous study [20], using HFFF-P16 as normal cells, demonstrated that methoxylated hydroxy flavones, including xanthomicrol, while lacking toxic activity on normal cells, could preferentially and selectively reduce the viable count of certain type of malignant cells. Jahaniani et al. (2005) had previously reported that mechanism of this discriminating antiproliferative effect might have been the induction of apoptotic, rather than necrotic, cell death in malignant cells [18]. The results of in vitro assays also showed that xanthomicrol potently decreased the viability of B16F10 melanoma cells. This data supports the presence of a direct antiproliferative effect of xanthomicrol on melanoma cells.

In the present study, we also conducted in vivo experiments to explore the antitumor activity of xanthomicrol and investigated the putative mechanisms of this potential effect. B16F10 cell xenograft is widely used to model melanoma in mice [24, 25]. Inoculation of mice with these cells produced tumors that were palpable after 15 days. Tumor development in animals in the thalidomide group was almost as rapid as the control group, which was suggestive of disease progression. In xanthomicrol-treated animals, however, tumor growth and progression was much slower compared with vehicle-treated animals. Measurement of tumor weight after tumor excision also showed a statistically significant retardation of tumor growth in xanthomicrol group compared with both thalidomide and vehicle.

Selective CD31 staining of endothelial cells lining the melanoma tumor vessels also confirmed the in vivo antiangiogenic effects of xanthomicrol. Notably, thalidomide failed to show efficient influence on MVD. This was apparently in contrast to its known anticancer and antiangiogenic effects [31]; however, this could be probably a result of the limitations of MVD to serve as a marker of antiangiogenic effect. Previous studies have also been unable to demonstrate a conclusive decrease in marrow angiogenesis following thalidomide therapy [34–36]. Therefore, as other studies also suggested, utilizing more sensitive predictors of angiogenesis such as quantification of VEGF mRNA expression by qRT-PCR is recommended [34]. These findings reveal that xanthomicrol may possess more potent anticancer effects than thalidomide, not only in inhibiting melanoma tumor growth and progression, but also in decreasing angiogenesis. The data obtained in our study are in agreement with the results of an ex vivo study, which showed that xanthomicrol had a greater direct antiangiogenic effect on endothelial cells compared with thalidomide [21]. The same study showed that antiangiogenic activity of xanthomicrol was probably mediated through inhibition of VEGF expression. VEGF is a powerful HIF-1*α*-regulated angiogenic factor secreted principally by tumor cells and tumor-infiltrating inflammatory cell types [8, 15]. In the present study, xanthomicrol was able to significantly inhibit HIF-1*α* and VEGF mRNA expression in tumor samples and reduce serum levels of VEGF. These results are consistent with clinical studies reporting that VEGF expression in tumor tissue specimens and sera of melanoma patients strongly correlates with angiogenic activity and stages of disease progression [7, 9]. Thus, it could be argued that direct antitumor effects with concomitant decrease in VEGF secretion via inhibition of HIF-1*α*/VEGF axis may account for the observed effect of xanthomicrol in reducing MVD. Moreover, results of clinical studies revealed that circulating VEGF is a useful predictive indicator of survival in various cancer patients [8, 9]. So, although we did not directly perform a survival study, decreased serum levels of VEGF following treatment with xanthomicrol supported the idea that it could have effectively prolonged the survival of animals in our melanoma tumor model.

Further molecular analysis of tumor tissues also revealed that xanthomicrol attenuated PI3K/Akt signaling pathway activity by decreasing Akt phosphorylation levels. The role of this pathway in regulating tumor growth and angiogenesis, via induction of HIF-1*α* and VEGF expression, is well-defined in melanoma and other cancer cell types [37]. Accordingly, the antiangiogenic property of xanthomicrol may be mediated, at least in part, through its inhibitory effects on Akt signaling pathway.

Bedongi et al. (2005) confirmed that synergism between Akt and HIF-1*α* signaling pathways is crucial for Akt-mediated melanoma development in vivo [14]. They also speculated this synergistic effect might be of great importance especially in the early stages of melanocyte transformation, while previously established tumor cells may induce angiogenesis independent of HIF-1*α*. This might explain how administration of xanthomicrol at early stages of melanoma development prevented initial tumor growth, and in some cases, this effect was observable until the end of the experiment. This may also explain why thalidomide, which showed an inhibitory trend on initial tumor growth, failed to display significant influence on tumor growth later on. This could be explained by its lack of effects on Akt and HIF-1*α*.

We also observed that incipient melanoma tumors in dormant state eventually progressed, and xanthomicrol was only able to retard, but not completely suppress, the tumor progression. Dankort et al. (2009) provided firm evidence showing that inhibitors of either PI3K or MAPK signaling pathways, used as single agents, notably prevented melanoma formation but did not completely eliminate melanoma cells [12]. They postulated the presence of long-lived melanoma cells that could remain alive even after prolonged blocking of the critical survival pathways required for melanoma cell proliferation. Thus, the inhibition of one of these two important pathways, namely, PI3K or MAPK, has basically a cytostatic rather than a cytotoxic effect on melanocytes. Consequently, this led to subsequent melanoma development after cessation of drug treatment. These results, together with the fact that xanthomicrol was only able to partially, but not completely, inhibit the Akt signaling pathway, best explained our observations in the late stages of tumor growth.

At doses up to 50 mg/kg, xanthomicrol has shown acceptable pharmacokinetic and safe toxicological profile in a previously performed study [22]. This current work has also shown a lack of overt toxicity attributable to xanthomicrol in an in vivo model. However, doses greater than 50 mg/kg were not achievable due to low solubility resulting in poor bioavailability of the compound. Thus, development of preparations using appropriate carrier systems, such as nano-carriers, to enhance its delivery is a prioritiy. This would not only improve the pharmacokinetic profile of xanthomicrol but also make it possible to use higher doses, which might lead to greater antitumor and antiangiogenic effects in vivo [16].

Cancer development is a multistep procedure, and different signaling pathways are involved [16]. As such, xanthomicrol appears to be an effective antiangiogenic candidate to be used as a part of a therapeutic regimen. Comparison of its effects with established RPK inhibitors like sunitinib or sorafenib will help determine its effectiveness in such a setting, especially as resistance mechanisms have limited the impact of current RPK inhibitors [3].

## 5. Conclusion

Taken together, our data provided experimental proof of concept for the benefit of xanthomicrol in subcutaneous mouse melanoma model. We also proposed that xanthomicrol might exert its antiangiogenic and antitumor effects, in part, via interfering with PI3K/Akt signaling pathway. This could subsequently lead to inhibition of HIF-1*α*/VEGF axis and VEGF secretion by tumor cells. However, further evaluation of xanthomicrol in other in vitro and in vivo tumor models and the associated signaling pathways is definitely required to provide a better understanding of the function of this potential antitumor agent in cancer therapy.

## Figures and Tables

**Table 1:** The primers used for gene expression analysis in tumor tissue.

Gene	Sequence	Product size (bp)
HIF-1*α*	Forward: 5′- TCCTGGAAACGAGTGAAAGGA -3′ (sense)	181
	Reverse: 5′- TTCTGCTGCCTTGTATGGGA -3′ (anti-sense)	

VEGFa	Forward: 5′-TTGTTCAGAGCGGAGAAAGC-3′ (sense)	193
	Reverse: 5′-GAGAGGTCTGGTTCCCGAAA-3′ (antisense)	

HPRT1	Forward: 5′ TCCCAGCGTCGTGATTAG-3′ (sense)	138
	Reverse: 5′- CGAGCAAGTCTTTCAGTCC-3′ (antisense)	

## Data Availability

No data were used to support this study.
